# Bladder Preservation with Primary Closure in an Adolescent Girl with Bladder Exstrophy

**DOI:** 10.4274/balkanmedj.2017.0077

**Published:** 2017-12-01

**Authors:** Yılmaz Aslan, Altuğ Tuncel, Ersin Köseoğlu, Çağdaş Şenel

**Affiliations:** 1 Department of Urology, University of Health Sciences, Ankara Numune Training and Research Hospital, Ankara, Turkey

## To The Editor,

**Figure 1:**
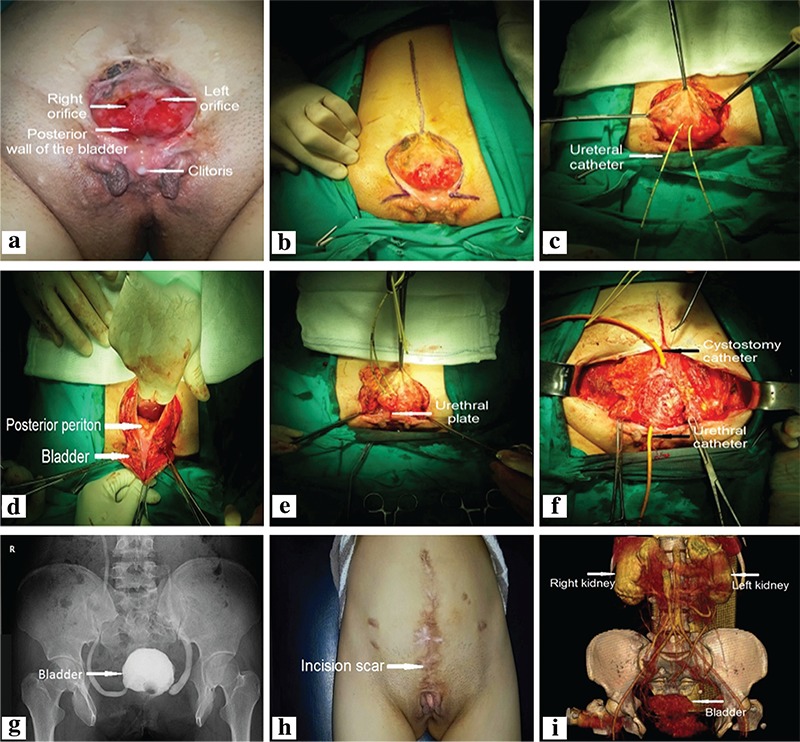
a-i. Pre-surgical view of the patient (a), skin incision (b), Ureteral catheterization of the ureters (c), the bladder and urethral plate were released from adjacent tissues (d, e), primary closure of the bladder (f), cystogram (g), healed abdominal wall defect at postoperative 16th months (h), computerized abdominal tomography at postoperative 16^th^ month (i).

A 16-year-old girl was diagnosed with bladder exstrophy in Afghanistan and referred to our clinic. A physical examination revealed that ureter orifices were observed at each side of the lower part of the bladder plate (Figure 1a). In Afghanistan, she did not accept cystectomy, augmentation or urinary diversion due to her country’s inadequate health care opportunities.

Informed consent was obtained from her family. In the supine position, we first performed an incision originating from superior of the umbilicus and enlarged through the adjacent part of the exstrophic bladder mucosa and extended to the paraexstrophic skin to the urethral plate (Figure 1b). We catheterized the ureters with 6 F ureteral catheters (Figure 1c). Secondly, the bladder was released from the posterior and lateral sides. Thirdly, we released the urethral plate from the adjacent tissue (Figure 1d, 1e). The fragile line forming the lateral inner wall of the bladder was excised for histopathological examination. Then, we closed the bladder, posterior urethra and muscle fibers anteriorly, leaving a space for a 12 F urethral catheter. Fourthly, we stripped the bifid labia medially. We put a cystostomy catheter into the bladder and two suction drains into the pelvic region. (Figure 1f). Lastly, we closed the abdominal skin. Pelvic osteotomy was not performed.

On postoperative day 7, we removed the ureteral catheters. On day 15, a cystogram was obtained and the cystostomy catheter was removed (Figure 1g). The urethral catheter was removed on postoperative day 21. Regarding continence, the patient was dry for 2-3 hrs with occasional stress incontinence, and voided through the uretra. The bladder capacity and average amount of residual urine volume on suprapubic ultrasonography was 100 and 30 cc, respectively. There was no malignancy on the histopathological examination of the specimen.

The patient came for follow-up at postoperative 16th month (Figure 1h). Abdominal computerized tomography showed a normal upper urinary tract and bladder (Figure 1i). Ureterosigmoidostomy was abandoned because of complications related to bladder exstrophy (1). Gulati et al. (2) reported two young adult females with bladder exstrophy who underwent cystectomy and a modified Mainz pouch. They claimed that the pouch has the advantages of achieving continence and greater capacity. Pathak et al. (3) treated four young adult male patients with classic bladder exstrophy and complete epispadias. All patients were continent after ileocystoplasty, bladder neck reconstruction and abdominal wall closure with flaps. Shoukry and Shoukry (4) reported their experience in five young adult classic bladder exstrophy patients. Three patients underwent primary repair (bladder closure, bladder neck reconstruction and epispadias repair). The surgeons performed augmentation iliocystoplasty in two of them. Another two patients underwent ureterosigmoidostomy, cystectomy and epispadias repair. They reported that one patient was continent and another two patients were continent with mild stress incontinence. In our case, primary closure of the bladder provided acceptable functional results without the need for additional surgery.

Bladder preservation seems to be safe and feasible in adolescent bladder exstrophy in poor sociocultural conditions. Our results should be confirmed with long-term follow-up in a larger number of patients.
